# Supplementary material for: The interactome of intact mitochondria by cross-linking mass spectrometry provides evidence for coexisting respiratory supercomplexes
Source: Mol Cell Proteomics. 2017 Dec 8;17(2):216–32. doi: 10.1074/mcp.RA117.000470 (PMC5795388; doi:10.1074/mcp.RA117.000470)
Supplement: Supplemental Data [file supp_RA117.000470_133922_0_supp_23978_fzffwf.zip › spectra_annotation/mito_spectra_annotation/59-1-10-1-17-1.pdf]

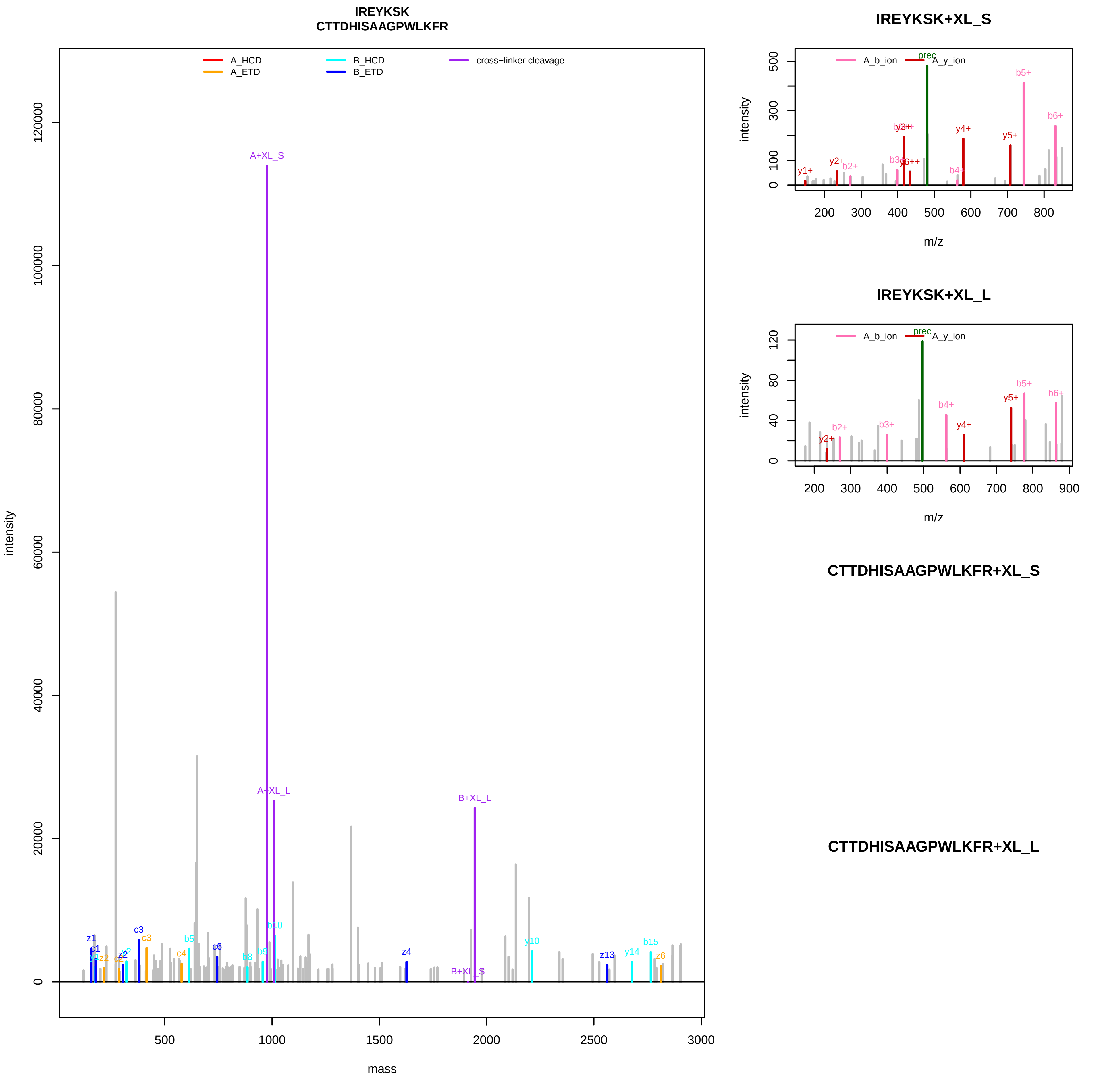
IREYKSK
CTTDHISAAGPWLKFR
A_HCD
A_ETD
B_HCD
B_ETD
cross−linker cleavage
A+XL_S
A+XL_L
B+XL_L
B+XL_S
z1
c1
z2
c3
c6
b5
c4
b8
b9
b10
z4
y10
z13
y14
b15
z6
intensity
mass
0
20000
40000
60000
80000
100000
120000
500
1000
1500
2000
2500
3000
IREYKSK+XL_S
A_b_ion
A_y_ion
prec
y1+
y2+
b2+
b3+
y3+
y6++
y4+
b4+
y5+
b5+
b6+
intensity
m/z
0
100
300
500
200
300
400
500
600
700
800
IREYKSK+XL_L
A_b_ion
A_y_ion
prec
y2+
b2+
b3+
b4+
y4+
y5+
b5+
b6+
intensity
m/z
0
40
80
120
200
300
400
500
600
700
800
900
CTTDHISAAGPWLKFR+XL_S
CTTDHISAAGPWLKFR+XL_L
